# Compatible Models of Carbon Content of Individual Trees on a *Cunninghamia lanceolata* Plantation in Fujian Province, China

**DOI:** 10.1371/journal.pone.0151527

**Published:** 2016-03-16

**Authors:** Lin Zhuo, Hong Tao, Hong Wei, Wu Chengzhen

**Affiliations:** 1 College of Forestry, Fujian Agriculture and Forestry University, Fuzhou City, Fujian Province, China; 2 Wuyi University, Wuyishan City, Fujian Province, China; DOE Pacific Northwest National Laboratory, UNITED STATES

## Abstract

We tried to establish compatible carbon content models of individual trees for a Chinese fir *(Cunninghamia lanceolata (Lamb*.*) Hook*.*)* plantation from Fujian province in southeast China. In general, compatibility requires that the sum of components equal the whole tree, meaning that the sum of percentages calculated from component equations should equal 100%. Thus, we used multiple approaches to simulate carbon content in boles, branches, foliage leaves, roots and the whole individual trees. The approaches included (i) single optimal fitting (SOF), (ii) nonlinear adjustment in proportion (NAP) and (iii) nonlinear seemingly unrelated regression (NSUR). These approaches were used in combination with variables relating diameter at breast height (D) and tree height (H), such as D, D^2^H, DH and D&H (where D&H means two separate variables in bivariate model). Power, exponential and polynomial functions were tested as well as a new general function model was proposed by this study. Weighted least squares regression models were employed to eliminate heteroscedasticity. Model performances were evaluated by using mean residuals, residual variance, mean square error and the determination coefficient. The results indicated that models with two dimensional variables (DH, D^2^H and D&H) were always superior to those with a single variable (D). The D&H variable combination was found to be the most useful predictor. Of all the approaches, SOF could establish a single optimal model separately, but there were deviations in estimating results due to existing incompatibilities, while NAP and NSUR could ensure predictions compatibility. Simultaneously, we found that the new general model had better accuracy than others. In conclusion, we recommend that the new general model be used to estimate carbon content for Chinese fir and considered for other vegetation types as well.

## Introduction

In recent years, many ecologists and policy makers have paid great attention to searching for ways to solve the problem of increasing concentrations of greenhouse gases, especially carbon dioxide (CO_2_) [1]. Due to the great potential of carbon sequestration, forests are viewed as one way to reduce atmospheric CO_2_ concentration [2]. The amount of carbon fixed in forest vegetation accounts for 2/3 of that in terrestrial ecosystems, making forest ecosystems a crucial carbon sink [3]. Thus, forests play an important role in preventing an increase in atmospheric CO_2_ concentrations and in the mitigation of global climatic changes. In this context, much research has already been conducted to estimate and predict the potential of carbon storage in different forest ecosystems [4–9]. However, it is complicated to estimate carbon storage due to the nonlinear and stochastic character of many vital processes, the complex interactions between different vegetation organs, and many limitations generated by incomplete and insufficient availability of data [10]. One appropriate approach for accurate simulation of carbon content is to develop models based on allometric relationships [11–12], and therefore, it is essential to explore more effective models for estimating carbon storage.

Carbon content of an individual plant refers to the part of carbon completely fixed in vegetation and is the net result of a series of uptake (e.g. photosynthesis) and loss processes (e.g. respiration). The amount of carbon content can be expressed as the total sum of carbon content of all components (including bole, branches, foliage leaves and roots) of an individual tree. Huang et al. [13] established carbon content models for individual trees of Chinese fir *(Cunninghamia lanceolata (Lamb*.*) Hook*.*)*. They used power function as a basic model form to construct models with some testability factors to create the carbon yield table for predicting carbon content of individual trees. However, the main deficiency of their research findings was only considering one basic model without compatibility for estimating carbon content, this weakness might have led to some deviations in practical application. In general, compatibility means that if one component is part of the tree, it is logical to expect the estimate of the component not to exceed the estimate of the whole tree. If a component is defined as the sum of a few subcomponents, its regression estimate value must be equal the sum of the regression estimates of all subcomponents. So like previous studies which were conducted based on allometric scaling theory [14–15], we decided to develop some equations to establish carbon content models and to provide the scientific rationale to guarantee compatibility of results.

The detailed objectives of this study are as follows: (i) to validate the performance of different carbon content models, especially incompatible and compatible ones; (ii) to analyse and compare different approaches for choosing the best way to solve the compatibility problem; and (iii) to propose a new general model for developing the form of carbon content model for individual trees.

## Methods

### Study area

**Fig 1:**
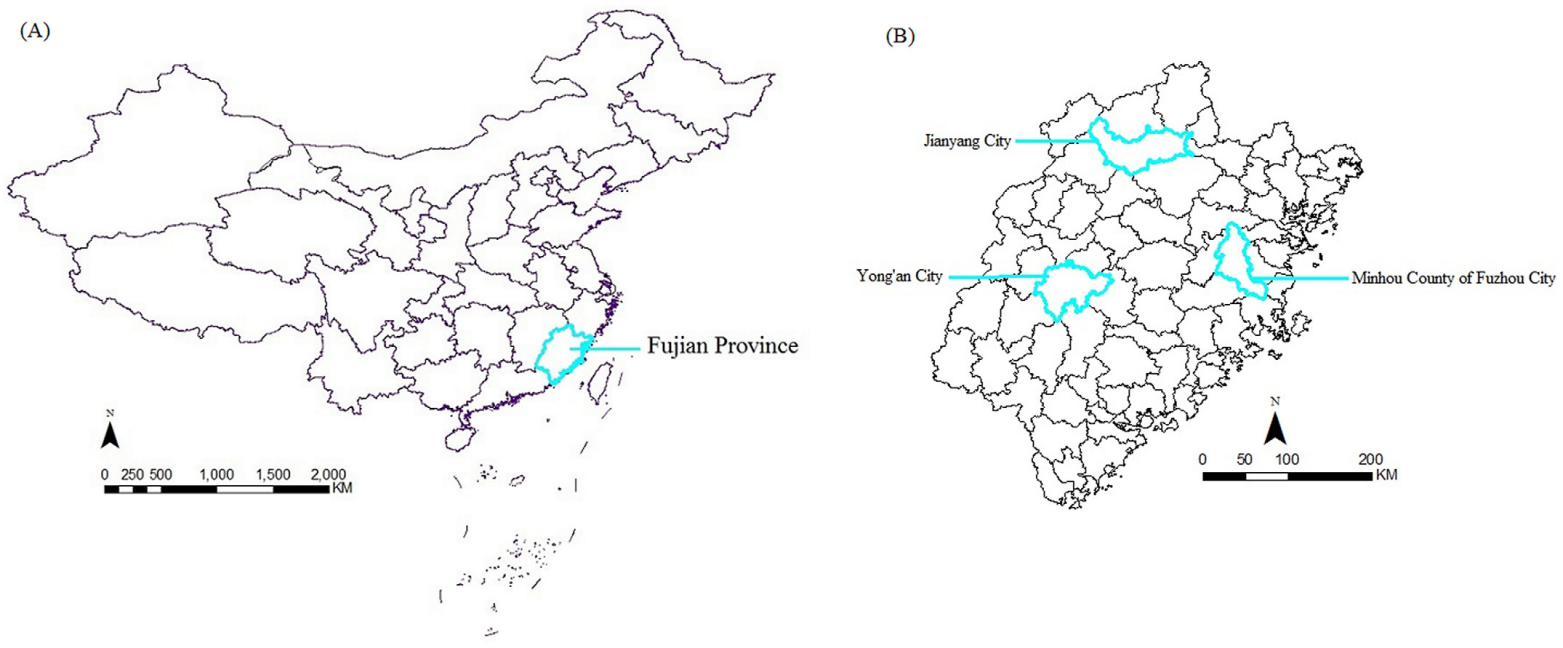
A map of Fujian Province showing the study locations. (A) The location of Fujian Province; (B) The locations of three study areas.

Fujian Province is located in southeast China. The geographical coordinates range between 23°30' N to 28°22' N, 115°50' E to 120°40' E (Fig 1A). It is situated near the Tropic of Cancer and is influenced by monsoon circulation such that the climate of this area is a subtropical marine monsoon. The province is characterized by abundant sunlight and rainfall. The annual average temperature is between 17°C and 21°C, while rainfall varies over a range of 1400 to 2000 mm. As one of the main production areas of Chinese fir (*Cunninghamia lanceolata*), according to the Eighth National Forest Resources Inventory of China (2009 to 2013), the forest area of Fujian Province is 8,012,700 hm^2^ with forest coverage that has reached 65.95%, hence ranking the first in the country. The principal soil types contain red, yellow and lateritic red earth. These climate and edaphic factors provide favourable environmental conditions for production of Chinese fir in Fujian province.

### Data resources

We conducted a stand survey from three Chinese fir plantation distribution areas (Fig 1B). These include (i) The central region (Jianyang City) which is found between 27°06' N to 27°43' N, 117°31' E to 118°38' E, (ii) The general region (Yong'an City) located at 25°33' N to 26°12' N, 115°56' E to 117°47' E and (iii) The edge region (Minhou County of Fuzhou City) located between 25°47' N to 26°37' N, 118°51' E to 119°25' E in Fujian Province [16]. The authority responsible for each study location: (i) Study areas of Jianyang City belong to Forestry Bureau of Jianyang City; (ii) Study areas of Yong'an City belong to Forestry Bureau of Yong'an City; (iii) Study areas of Minhou County of Fuzhou City belong to Baisha State-owned Forest farm of Minhou County. All studies in these fields did not involve endangered or protected species and all activities for research were permitted by the authority responsible for each study location. Based on site index, stand age, elevation, slope gradient, slope aspect and slope position, we used a quadratic orthogonal rotational combining design [17–18] to lay out 54 plots (18 plots in each region, for details, see S1 Table). Some key points of sample plots included: stand age from 6 to 12 years, stand density from 1000 to 4000 plants/hm^2^, site index from 6 to 28 m, stand average diameter at breast height (DBH, D) from 7.1 to 30.8 cm, and stand average tree height from 8.5 to 23.8 m. Based on the characteristics of each diameter class, we chose one sample tree per plot to investigate the biomass.

After the tree was felled, we excavated the soil to get roots by using the whole roots digging method [19]. The roots included large sized roots (2–5 cm), middle sized roots (0.5–2 cm), small sized roots (0.2–0.5 cm), fine roots (< 0.2 cm) and stump roots (> 5cm). Then the whole plant parts were obtained. We first measured and recorded the fresh weights of bole, branches, foliage leaves and roots of each sample and took some subsamples from each component. In the laboratory, we processed the subsamples, and then all samples were oven dried at 105°C for 2 hour once a time until a constant weight for each sample was obtained. Then oven dry weights were calculated for bole, branches, foliage leaves and roots. After drying, grinding, sieving and heating with potassium dichromate (K_2_Cr_2_O_7_), we obtained the carbon content of the subsamples for each component. Then, we calculated the average ratios of carbon content (kg per 1 kg weight) from different regions: boles = 0.5229(s.d. = 0.0059), branches = 0.5008 (s.d. = 0.0055), foliage leaves = 0.4794(s.d. = 0.0057), roots = 0.5048 (s.d. = 0.0062), aboveground = 0.516 (s.d. = 0.0058) and the whole tree = 0.514 (s.d. = 0.0057). Next, we multiplied the biomass of boles, branches, foliage leaves and roots by the corresponding ratio of carbon content. Finally, we obtained the carbon content of each component of the sample trees as model sample data to establish models.

Simultaneously, we collected validation data from previous publications on Chinese fir biomass in Fujian Province [19–27] and chose 27 sample trees that had complete measured data, including D, H and biomass of each component. However, because some measurement methods were inconsistent, we decided to make the following adaptations before using them: (i) We classified component data into four parts: bole (including bark), branches, foliage leaves and roots. Whenever we found existing data (from literatures) on aboveground and the whole biomass, we used them. Otherwise, we assumed that the aboveground biomass is the sum of bole, branches and leaves biomass, and the sum of aboveground and roots biomass was the whole tree biomass. (ii) Because we needed to validate the predictive abilities of carbon content models, we converted biomass data into carbon content. Assuming that the average ratios of carbon content calculated in this study were accurate and applicable, then we used these ratios to obtain carbon content of validation samples. Table 1 shows some statistics for the modelling and validation data (details of modelling and validation samples are in S2 and S3 Tables, respectively).

**Table 1:** Statistics of modelling and validation samples data.

	Modelling samples (N = 54)				Validation samples (N = 27)			
	Minimum	Maximum	Mean	Standard deviation	Minimum	Maximum	Mean	Standard deviation
**D(cm)**	8.40	28.70	16.45	5.42	5.00	21.00	14.17	5.34
**H(m)**	10.60	22.50	14.59	2.42	6.22	22.10	13.39	5.13
**W**_**1**_**(kg)**	10.89	204.44	58.09	42.96	3.30	115.29	46.71	35.85
**W**_**2**_**(kg)**	8.48	175.13	49.11	37.88	2.66	98.50	38.12	29.79
**W**_**3**_**(kg)**	6.31	153.93	41.22	33.36	1.31	85.22	31.16	26.35
**W**_**4**_**(kg)**	1.42	12.48	4.30	2.53	0.91	7.32	3.65	1.78
**W**_**5**_**(kg)**	0.75	9.42	3.59	2.18	0.38	7.64	3.32	1.95
**W**_**6**_**(kg)**	2.42	29.31	10.69	6.69	0.64	22.14	8.57	6.26

D represented diameter at breast height; H represented tree height; W_1_, W_2_, W_3_, W_4_, W_5_, W_6_ represented carbon content of the whole tree, aboveground, bole, branches, foliage leaves and roots, respectively.

### Basic models and Variable selection

In general, to obtain the carbon content of plants, scholars usually establish biomass models at first, and then use conversion factors to obtain carbon content results [28–30]. In this study, we directly measured the carbon content of each tree component (i.e., bole, branches, foliage leaves and roots). We applied power, exponential and polynomial functions (which were used to build models for biomass [14, 31–34]) and a new general model proposed by this study to establish carbon content model of individual trees. We used F-test decider [35] to determine the maximum order of polynomial function that was equivalent to “2” so that a polynomial function form was a quadratic polynomial.

According to the findings from using different variables to estimate biomass in previous studies, it is recommended to use D which is easily measured and is the best parameter for estimating the biomass of each component [36–37]. Moreover, based on different types of models reported by previous researches [38–39], the models with two dimensional variables, D and H (tree height), were much more strongly correlated with biomass than those with only one variable. For that reason, we focused on many different combinations between D and H, including D, DH, D^2^H and D&H (D&H meant two separate variables in bivariate model) as variables to establish carbon content models.

We assumed that *f*_i_(*x*) (*i* = 1, …, 6) represented carbon content equations of the whole tree, aboveground, for bole, branches, foliage leaves and roots. The different basic models were as follows:

Single variable model with different variable combinations, including D, DH and D^2^H:

$$f_{\text{i}}(x)=a\cdot x^{b}$$ (1)

$$f_{\text{i}}(x)=c\cdot e^{d\cdot x}$$ (2)

$$f_{\text{i}}(x)=f\text{+}g\cdot x\text{+}h\cdot x^{2}$$ (3)

Bivariate model with variables D&H:

$$f_{\text{i}}(x,\text{y})=a\cdot x^{b}\cdot y^{c}$$ (4)

$$f_{\text{i}}(x,y)=d\cdot e^{f\cdot x\cdot y}$$ (5)

$$f_{\text{i}}(x,y)={\left(g\cdot x\text{+}h\cdot y+j\right)}^{2}$$ (6)

where by Eqs 1 and 4 represented a form of a power function; Eqs 2 and 5 represented a form of an exponential function; Eqs 3 and 6 represented a form of a polynomial function; e is the base of natural logarithms; *a*, *b*, *c*, *d*, *f*, *g*, *h*, *j* were parameters.

In this study, we first proposed a new general model as a basic model, and the form of a single variable model could be written as:

$$f_{\text{i}}(x)={\left(\alpha \cdot x^{\beta }+\gamma \cdot e^{\eta \cdot x}\right)}^{\theta }$$ (7)

where by *α*, *β*, *γ*, *η*, *θ* were parameters, and when *η* = 0, *θ* = 1, Eq 7 could be transformed into a form of a power function (Eq 1); when *β* = 0, *θ* = 1, Eq 7 could be transformed r into a form of an exponential function (Eq 2); when *β* = 1, *η* = 0, and Eq 7 could be transformed into a form of a polynomial function (Eq 3). Hence, we considered Eq 7 as the general model that contained various forms of functions, including power, exponential and polynomial functions. Similarly, the general bivariate model was:

$$f_{\text{i}}(x,y)={\left(\alpha \cdot x^{\beta }\cdot y^{\varepsilon }+\gamma \cdot e^{\eta \cdot x\cdot y}\right)}^{\theta }$$ (8)

where by *α*, *β*, *γ*, *η*, *θ*, *ε* were parameters.

To compare and evaluate performance of different carbon content models, we used four fit statistics: mean residuals (MR), residual variance (RV), mean square error (MSE), and determination coefficient (R^2^). They were calculated using the following equations:

$$MR=\sum (y_{i}-{\hat{y}}_{i})/\text{N}$$ (9)

$$RV=\sum {\left(y_{i}-{\hat{y}}_{i}\right)}^{2}/(\text{N}-1)$$ (10)

$$MSE=\sqrt{MR^{2}+RV}$$ (11)

$$R^{2}=1-{\sum \left({\hat{y}}_{i}-y_{i}\right)}^{2}/{\sum \left(y_{i}-\bar{y}\right)}^{2}$$ (12)

where by *y*_*i*_ represented observed values of the i^th^ sample tree; ${\hat{y}}_{i}$ represented estimated values of the i^th^ sample tree; $\bar{y}$ represented arithmetic mean of all observed values; *N* represented number of sample trees.

### Single optimal fitting

Single optimal fitting (SOF) used the traditional least square method to establish a single optimal regression model. This approach fitted equations separately based on the respective carbon content data of each component of individual trees. Consequently, the sum of carbon content predictions from the separate models of each component might not equal carbon prediction from the whole tree carbon content model. The model was expressed as follows:

$$W_{i}=f_{\text{i}}(x)+\varepsilon_{\text{i}}(i=1,\cdot \cdot \cdot ,6)$$ (13)

where by *f*_i_(*x*) represented a single optimal regression model of each component; *W*_*i*_ (*i* = 1, …, 6) represented carbon content of the whole tree, aboveground, bole, branches, foliage leaves and roots respectively; ε_i_ represented random error of each component.

### Compatible estimation approach

#### Nonlinear adjustment in proportion

According to the concept of compatibility, to satisfy that the sum of each component was equal to the whole tree, it was important to ensure that the sum of each component proportion was 100%. Following this logic, Tang et al. [40] adopted the nonlinear adjustment in proportion (NAP) to guarantee compatibility. This approach had two alternative procedures in common use; one was directly controlling the total using proportional functions (NAPI), and the other was jointly controlling from level to level by ratio functions (NAPII).

The procedure of NAP I was to directly assign the whole tree amount into bole, branches, foliage leaves and roots by each respective proportion, and each component function was expressed as follows:

$$W_{3}=\frac{f_{3}(x)}{f_{3}(x)+f_{4}(x)+f_{5}(x)+f_{6}(x)}W_{1}+\varepsilon_{3}$$ (14)

$$W_{4}=\frac{f_{4}(x)}{f_{3}(x)+f_{4}(x)+f_{5}(x)+f_{6}(x)}W_{1}+\varepsilon_{4}$$ (15)

$$W_{5}=\frac{f_{5}(x)}{f_{3}(x)+f_{4}(x)+f_{5}(x)+f_{6}(x)}W_{1}+\varepsilon_{5}$$ (16)

$$W_{6}=\frac{f_{6}(x)}{f_{3}(x)+f_{4}(x)+f_{5}(x)+f_{6}(x)}W_{1}+\varepsilon_{6}$$ (17)

$$W_{1}=f_{1}(x)+\varepsilon_{1}$$ (18)

where by *f*_1_(*x*) represented a single optimal regression model of the whole tree carbon content; and *f*_3_(*x*), *f*_4_(*x*), *f*_5_(*x*), *f*_6_(*x*) represented a single optimal regression model of carbon content of bole, branches, foliage leaves and roots, respectively.

The NAP II firstly assigned the carbon content of the whole tree into two parts by proportion, including aboveground and roots. Then, the aboveground carbon content was divided into bole, branches and foliage leaves. This approach not only guaranteed that the sum of the component predictions was equal to the whole tree but also that the bole, branches and foliage leaves predictions would sum up to the aboveground prediction. Detail models were expressed by the following equations:

$$W_{2}=\frac{f_{2}(x)}{f_{2}(x)+f_{6}(x)}W_{1}+\varepsilon_{2}$$ (19)

$$W_{3}=\frac{f_{3}(x)}{f_{3}(x)+f_{4}(x)+f_{5}(x)}\frac{f_{2}(x)}{f_{2}(x)+f_{6}(x)}W_{1}+\varepsilon_{3}$$ (20)

$$W_{4}=\frac{f_{4}(x)}{f_{3}(x)+f_{4}(x)+f_{5}(x)}\frac{f_{2}(x)}{f_{2}(x)+f_{6}(x)}W_{1}+\varepsilon_{4}$$ (21)

$$W_{5}=\frac{f_{5}(x)}{f_{3}(x)+f_{4}(x)+f_{5}(x)}\frac{f_{2}(x)}{f_{2}(x)+f_{6}(x)}W_{1}+\varepsilon_{5}$$ (22)

$$W_{6}=\frac{f_{6}(x)}{f_{2}(x)+f_{6}(x)}W_{1}+\varepsilon_{6}$$ (23)

$$W_{1}=f_{1}(x)+\varepsilon_{1}$$ (24)

where by *f*_2_(*x*) represented the single optimal regression model of aboveground carbon content.

#### Nonlinear seemingly unrelated regression

Parresol [41] presented an approach of nonlinear seemingly unrelated regression (NSUR) to solve the question on the addition of nonlinear biomass equations. The methodology considered the correlation among each component and guaranteed the compatibility between the whole tree and each component. In this study, we combined NSUR and the modified simplex method (MSM) [42] to estimate parameters. The equation for applying NSUR in this study was described as follows:

$$\left\{ \begin{array}{l} W_{3}=f_{3}(x)+\varepsilon_{3}\\ W_{4}=f_{4}(x)+\varepsilon_{4}\\ W_{5}=f_{5}(x)+\varepsilon_{5}\\ W_{6}=f_{6}(x)+\varepsilon_{6}\\ W_{2}=f_{2}(x)=f_{3}(x)+f_{4}(x)+f_{5}(x)+\varepsilon_{2}\\ W_{1}=f_{1}(x)=f_{3}(x)+f_{4}(x)+f_{5}(x)+f_{6}(x)+\varepsilon_{1} \end{array}\right.$$ (25)

### Heteroscedasticity removal

Normally, carbon content data exhibits heteroscedasticity; the error variance is not constant across all observations. Therefore, we removed heteroscedasticity before estimating parameters. The usual methods employed to remedy heteroscedasticity are the logarithmic transformation and weighted least squares regression [43]. In this study, we used weighted least squares regression to solve the heteroscedasticity problem based on regression analysis between variable values and residual square values of a single optimal regression model of each component [44]. According to Akaike Information Criterion (AIC) index, the optimum form of weighted function was a power function [15, 45], and the equation was as follows:

$$\psi_{\text{i}}(x)=1/{\text{g}}_{\text{i}}{\left(x\right)}^{2}$$ (26)

where by the forms of g_i_(*x*) were $D^{a_{1}}$,${\left(DH\right)}^{a_{2}}$,${\left(DH^{2}\right)}^{a_{3}}$ and $D^{a_{4}}H^{a_{5}}$ when setting different variables, respectively; *a*_1_, *a*_2_, *a*_3_, *a*_4_, *a*_5_ were parameters; i represented different components.

### Software

All results were calculated by using DPS 7.05 [46].

## Results

According to Eq 26, weighted functions of different variables were calculated (see details in S4, S5, S6 and S7 Tables). After removing heteroscedasticity by using weighted functions, we used different variables to establish carbon content models of each component and the whole tree for a modelling sample. Due to space limitations, we only listed optimal estimation results of different variables in Table 2, and the detail comparison evaluation indices of four basic models in S8, S9, S10 and S11 Tables.

**Table 2:** The evaluation indices of optimal estimation results by using four basic models with different variables.

Component	Variable	Model	R^2^	Mean Residual	Residual Variance	Mean Square Error
**Bole**	D	Eq 7	0.9705	-0.1155	32.8280	5.7307
	DH	Eq 3	0.9849	-0.0008	16.8416	4.1038
	D^2^H ^a^	Eq 1	0.9922	0.1258	8.6308	2.9405
	D & H ^b^	Eq 8	0.9929	-0.0958	7.9379	2.8191
**Branches**	D	Eq 7	0.8916	-0.0017	0.6919	0.8318
	DH	Eq 3	0.9127	-0.0008	0.5573	0.7465
	D^2^H	Eq 7	0.9155	0.0001	0.5391	0.7342
	D & H	Eq 8	0.9167	0.0003	0.5320	0.7294
**Foliage leaves**	D	Eq 7	0.8938	-0.0105	0.5060	0.7114
	DH	Eq 7	0.9308	0.0001	0.3298	0.5743
	D^2^H ^a^	Eq 1	0.9256	-0.0121	0.3545	0.5955
	D & H	Eq 8	0.9315	-1.6667E-05	0.3264	0.5713
**Roots**	D	Eq 7	0.9517	-0.0095	2.1635	1.4709
	DH	Eq 3	0.9408	0.0008	2.6514	1.6283
	D^2^H ^a^	Eq 1	0.9559	-0.0388	1.9724	1.4050
	D & H	Eq 6	0.9598	-0.0028	1.7999	1.3416
**Aboveground**	D	Eq 7	0.9732	-0.1557	38.4567	6.2033
	DH	Eq 3	0.9854	-0.0008	20.9060	4.5723
	D^2^H	Eq 3	0.9921	-9.26E-06	11.3360	3.3669
	D & H	Eq 8	0.9928	-0.0128	10.45408	3.2333
**Whole tree**	D	Eq 7	0.9771	-0.1597	45.1548	6.7216
	DH	Eq 3	0.9856	-0.0008	28.3301	5.3226
	D^2^H ^a^	Eq 1	0.9939	0.1256	11.9725	3.4624
	D & H	Eq 8	0.9944	-0.0579	10.9877	0.9944

Eq 1 (Eq 4), Eq 3 (Eq 6) and Eq 7 (Eq 8) represented a power function (Eq 4 was bivariate), a polynomial function (Eq 6 was bivariate) and the general model (Eq 8 was bivariate), respectively.

^a^ Under the condition of rounding four decimal places, the results were almost the same between power function (Eq 1) and general model (Eq 7) for bole, foliage leaves, roots and the whole tree models with variable D^2^H (in S10 Table). Considering Eq 1 had less parameters, we considered Eq 1 was optimal due to more convenient application in practice.

^b^ Under the condition of rounding four decimal places, the R^2^ of a power function (Eq 4) and the general model (Eq 8) were same, but the general model had lower RV and MSE, so we considered Eq 8 was optimal (in S11 Table).

**Fig 2:**
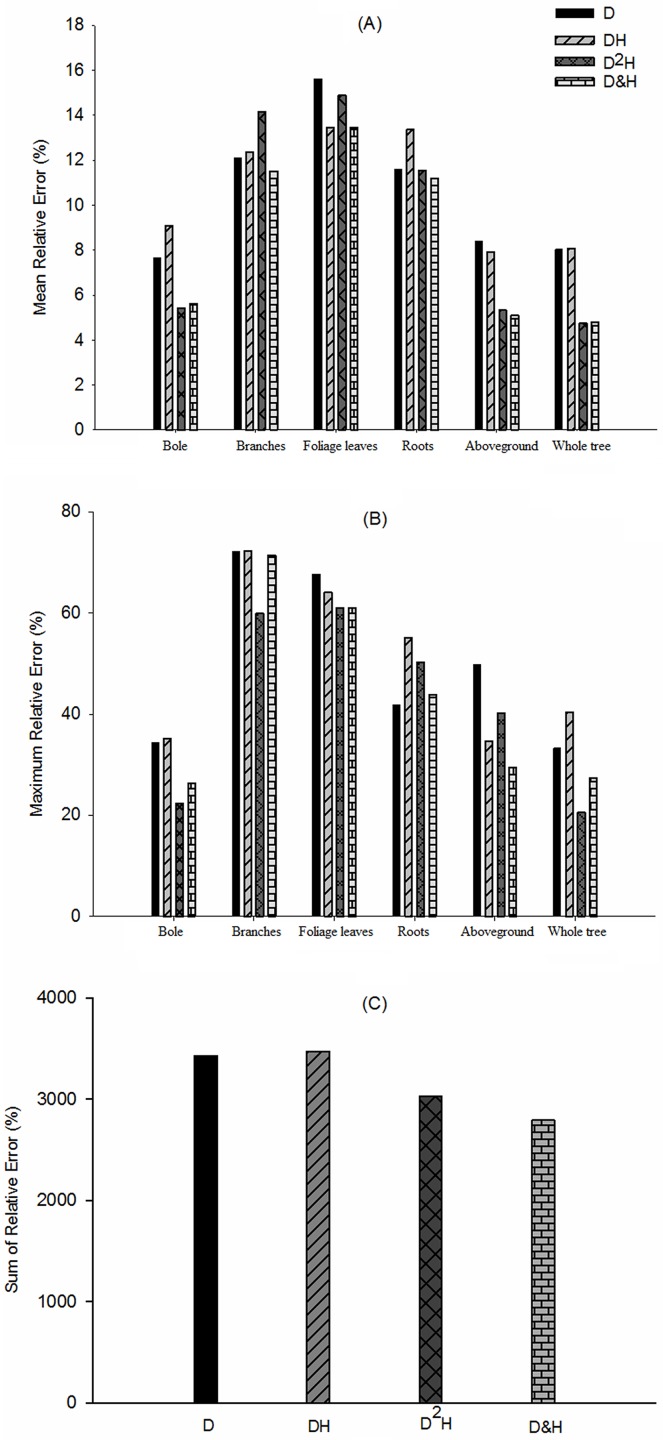
The relative errors of predictions for different variables. (A) Mean relative error for each component; (B) Maximum relative error for each component; (C) Sum of all prediction relative errors for different variables.

We found (Table 2) that the best basic model to estimate carbon content was the general model (Eq 7). This model had the highest R^2^ as well as the lowest RV and MSE for each component and the whole tree, when only D as variable was used (S8 Table). However, there were different results when a combination DH as a variable was used. Except for foliage leaves, the results revealed that the best model form was a polynomial (quadratic polynomial, Eq 3). Moreover, using Duncan’s multiple range tests, we found that there was a significant difference (at 0.01 significance level) between Eq 2 and the other three models (Eqs 1, 3 and 7) with variable DH (S9 Table). Interestingly, under the condition of rounding to four decimal places and using D^2^H as the combination variable to establish models (S10 Table), the evaluation indices of a power function and the general model were almost the same except for branches (whose optimal model was the general model, Eq 7) and aboveground (whose optimal model was polynomial, Eq 3). Meanwhile, Fig 2 compares relative errors of predictions by using optimal models with different variable combinations (based on Table 2). Fig 2A and 2B show the mean relative and maximum relative errors for each component with different variables respectively. Fig 2C compares the sum of all prediction relative errors for different variables. The bivariate models using D&H as variables had the best fitting abilities for all components and the whole tree (see Table 2 and Fig 2). Likewise, by comparing all optimal estimation results, the optimal form was the bivariate general model (Eq 8) except for roots, for which a polynomial function (Eq 6) had higher precision than other forms.

D represented diameter height at breast and H represented tree height.

The formula of relative error was $(\frac{\left|estimation-observation\right|}{observation})\times 100\%$

Based on the results listed in Table 2, the optimal basic model is combined with D&H to establish compatible models as follows: Eq 4-power function (bole), Eq 6-polynomial function (roots), Eq 8-bivariate general model (branches, foliage leaves, aboveground and the whole tree). According to the formulas from Eq 14 to Eq 25, we obtained compatible models using NAP I, NAP II and NSUR, the results of which are shown in Table 3. Through Duncan’s multiple range tests, there was no significant difference (at 0.01 significance level) among different approaches. The accuracies of different compatible approaches were characterized by this sequence: whole tree > aboveground > bole > roots > foliage leaves and branches. Furthermore, the performance of compatible models was relatively good. The precision of aboveground (0.9932) and the whole tree (0.9947) by using NSUR were even better than the results by SOF. The highest R^2^ by SOF for aboveground and the whole tree were 0.9928 and 0.9944, respectively (Table 2). As for the models of each component, we found that the results by NAP approach were slightly better than results by NSUR.

**Table 3:** Comparisons of precision and stability of different compatible estimation approaches.

Approach	Component ^a^	R^2^	Mean Residual	Residual Variance	Mean Square Error
**NSUR**	Bole	0.9928	-0.0015	7.9643	2.8221
	Branches	0.8858	-0.0291	0.7293	0.8545
	Foliage leaves	0.8856	0.0235	0.5454	0.7389
	Roots	0.9594	-0.0024	1.8193	1.3488
	Aboveground	0.9932	0.1712	9.7556	3.1281
	Whole tree	0.9947	-0.0096	10.3847	3.2225
**NAP I**	Bole	0.9929	0.0103	7.5465	2.7471
	Branches	0.9157	-0.0639	0.5378	0.7362
	Foliage leaves	0.9321	-0.0081	0.3234	0.5687
	Roots	0.9595	-0.0221	1.8112	1.3460
	Aboveground ^b^	-	-	-	-
	Whole tree	0.9944	-0.0096	10.3847	3.2225
**NAP II**	Bole	0.9929	0.0627	7.9544	2.8211
	Branches	0.9156	-0.0680	0.5387	0.7371
	Foliage leaves	0.9321	-0.0107	0.3235	0.5688
	Roots	0.9598	0.0084	1.8018	1.3423
	Aboveground	0.9931	-0.0921	9.9564	3.1567
	Whole tree	0.9944	-0.0096	10.3847	3.2225

Through Duncan’s multiple range tests, there was no significant difference (at 0.01 significant level) among different approaches.

^a^ The optimal basic models for each component and the whole tree were as follows: Eq 4- a power function for bole, Eq 6-a polynomial function for roots, and Eq 8- the bivariate general model for branches, foliage leaves, aboveground and the whole tree. Specifically, we used Eq 4 as the optimal model for bole in order to reduce estimation parameters on the condition of same R^2^ between Eqs 4 and 8.

^b^ On the basis of the procedure of NAP I, it did not involve aboveground in processing of estimating compatible models, so the values of evaluation indices were null.

To compare the predictive abilities between compatible and incompatible models, we estimated the parameters of the optimal models using SOF (incompatible models) and compatible models by NSUR (Tables 2 and 3). Then, we used these models to simulate validation data and compute various evaluation indices (Table 4). The results of T-test revealed that there were no significant differences (at 0.01 significance level) between SOF and NSUR. As a complete unit, predictive effects of model by NSUR were superior to those by SOF. However, whether by SOF or by NSUR, the predictive accuracies of branches and foliage leaves were relatively low (at approximately 0.80). Additionally, the accuracy of the aboveground carbon content predicted by SOF (0.8720) was obviously lower as compared to the result simulated by NSUR (0.9548). For the carbon content of bole and roots as well as the whole tree, we found that there were no significance differences between the two approaches. The relative errors of some predictive values had large deviations. The maximum deviations of the two approaches reached 95.80% (by SOF) and 131.24% (by NSUR). In terms of average relative error, the values of branches and foliage leaves were greater than 20%. However, there were two outliers (detected by triple standard deviation approach, at 0.01 significant level) in predictive value for branches by NSUR. After elimination of these two outliers, the maximum relative error and the mean relative error for branches by NSUR changed to 67.27% and 16.92%, respectively. In addition, other component predictive values were relatively good, including the bole, roots, aboveground and the whole tree. Although R^2^ (0.9296%) and mean relative errors (11.39%) were acceptable for predicting the carbon content of roots by using SOF, the results showed that fourteen(14) predicting relative error values were greater than 20% (Table 4). So we considered that the effects of carbon content prediction on roots by SOF were not very good.

**Table 4:** Results of simulating validation data by SOF and NSUR.

Approach	Component	Model	R^2^	Relative Error			
				Minimum	Maximum	Mean	N(RE≥20%)
**SOF**	Bole ^a^	W_3_ = 0.0101•D^1.8635^•H^1.093^	0.9503	0.43%	29.95%	12.36%	3
	Branches	W_4_ = (0.0895•D^0.5532^•H^0.2987^+0.5295•exp(0.0008•D•H))^3.019^	0.7836	4.19%	35.33%	20.02%	15
	Foliage leaves	W_5_ = (-7.0756•D^-0.0152^•H^-0.0134^+7.6321•exp((-2.85E-06)•D•H))^17.4524^	0.8104	0.57%	95.80%	35.47%	14
	Roots	W_6_ = (0.1535•D+0.0589•H-0.2529)^2^	0.9296	1.09%	28.35%	11.39%	14
	Aboveground	W_2_ = (0.0417•D^1.3489^•H^0.8006^+0.7488•exp(-0.0031•D•H))^1.3516^	0.8720	0.20%	42.84%	15.11%	5
	Whole tree	W_1_ = (0.0232•D^1.8278^•H^1.0188^+1.4803•exp(-0.0028•D•H))^0.9650^	0.9610	0.50%	28.75%	8.95%	3
**NSUR**	bole	W_3_ = 0.0101•D^1.852^•H^1.1077^	0.9503	0.50%	30.92%	12.81%	4
	Branches ^b^	W_4_ = (-0.0015•D^-5.0771^•H^6.4394^+1.2283•exp(0.0007•D•H))^4.0968^	0.8415	0.80%	131.24%/67.27%	24.22%/16.92%	7/5
	Foliage leaves	W_5_ = (-0.2287•D^-5.3579^•H^4.8691^+1.1662•exp(0.0004•D•H))^5.7851^	0.8022	0.30%	73.00%	21.00%	13
	Roots	W_6_ = (0.1493•D+0.0698•H-0.3433)^2^	0.9310	2.39%	29.46%	11.04%	4
	Aboveground	W_2_ = W_3_+W_4_+W_5_	0.9548	0.22%	53.89%	14.61%	7
	Whole tree	W_1_ = W_3_+W_4_+W_5_+W_6_	0.9618	0.21%	45.19%	11.82%	4

Through T-test, there was no significant difference (at 0.01 significant level) between SOF and NSUR.

^a^ As results of performances of a power function were almost the same as the general model, we decided to use power function as optimal models by SOF, in order to reduce estimation parameters.

^b^ Through detecting by triple standard deviation approach, there were two outliers among results of relative error for branches (131.24% and 99.83%), so we recalculated results after elimination of these two values (the latter ones of Maximum, Mean and N of Relative Error).

The formula of relative error was $(\frac{\left|estimation-observation\right|}{observation})\times 100\%$

## Discussion

### Necessity of establishing compatible model

Compatibility requires that the sum of components equal the whole tree, so the sum of percentages calculated from component equations must equal 100%. But using SOF to construct models would lead to greater deviations in predicting the sum of the carbon content of each component and that of the whole tree. Some examples were listed in Table 5 to illustrate incompatibility for modelling and validation samples by using SOF. It showed that incompatible errors existed in estimated values for both the whole tree and aboveground carbon content while the magnitude of errors was unknown. If these estimated results were applied in a practical situation, it might lead to confusions and mistakes. Hence, it was necessary to solve the compatibility problem when estimating carbon content. Through comparisons in this study, we found that compatible models took full account of mathematical relations and inherent correlations between the whole tree and each component. These models which were constructed by using NAP and NSUR guaranteed that the sum of each component’s carbon content was equivalent to that of the whole tree in the range of systematic errors permitted. Additionally, predictive abilities of the compatible models were even better than incompatible models to some extent (Table 4). So we concluded that it was better to establish compatible models for simulating carbon content of individual trees.

**Table 5:** Examples of incompatibility for modelling and validation samples.

Sample	D(cm)	H(m)	△1(kg)	△2	△3(kg)	△4	△5(kg)	△6
**Modelling sample**	8.6	11.4	0.2035	1.47%	0.3254	2.97%	-0.1220	-0.88%
	14.9	14.65	-0.5600	-1.27%	-0.7030	-1.97%	0.1430	0.32%
	17.6	15.6	-0.9523	-1.52%	-1.2564	-2.47%	0.3041	0.49%
	22.4	15.04	-1.6080	-1.75%	-1.7354	-2.30%	0.1274	0.14%
	25.5	18.2	-2.8815	-2.07%	-3.2128	-2.76%	0.3313	0.24%
	28.7	22.5	-5.6395	-2.67%	-5.5784	-3.08%	-0.0611	-0.03%
**Validation sample**	6.17	7.76	0.7286	10.90%	1.2486	21.75%	-0.5200	-7.78%
	13.3	10.2	0.1287	0.50%	2.2811	10.26%	-2.1526	-8.35%
	20.18	22.1	-0.2639	-0.23%	11.8152	11.10%	-12.0790	-10.83%

D represented diameter at breast height; H represented tree height; △1 = W_1_-W_3_-W_4_-W_5_-W_6_; △2 = (△1/ W_1_) ×100%;

△3 = W_2_-W_3_-W_4_-W_5_; △4 = (△2/ W_2_)×100%; △5 = W_1_-W_2_-W_6_; △6 = (△4/ W_1_) ×100%; W_1_, W_2_, W_3_, W_4_, W_5_, W_6_ represented carbon content of the whole tree, aboveground, bole, branches, foliage leaves and roots of individual trees, respectively.

### Comparisons of different fitting approaches

Although we could obtain single optimal models by using SOF, these models did not guarantee compatibility. However, NAP and NSUR guaranteed the property of carbon content additivity among the components of the tree and the whole tree (Table 3). Nonetheless, there were still some flaws in calculation for these two compatible estimation approaches. NAP used single optimal models calculated by SOF to determine the proportion coefficient for each component, and parameters of single optimal models were estimated using the traditional least square method. Due to the parameters being calculated without considering compatibility, there were prediction errors in the results. When using NAP, these errors were passed down as a process of grading adjustment, and the final results of the compatible models of each component were no longer optimal [15].

Referring to relevant literature [41], NSUR was based on a nonlinear joint-generalized regression with parameter restrictions. In this context, NSUR permitted each component model to use its own weighting function for removing heteroscedasticity and obtain a lower variance for the aboveground and the whole tree carbon content models. Unlike the linear model, a nonlinear estimation would pose many challenges [41], such as the initial parameters estimation needing to be specified. If we could not obtain appropriate starting values, the computing time would be long and a nearly flat gradient could lead to slow convergence. Various methods could be available for obtaining starting values such as experience and estimating linearization by transformation with ordinary least squares. If these issues were not a concern, it would be preferable to use NSUR to establish compatible models which had higher accuracies and smaller estimation errors in theory, by considering overall precision and stability.

However, we noticed that according to compatible results in Table 3, there were some small contradictions between this study and previous literature [15,40], which concluded that the precision of NSUR was better than NAP. Except for the aboveground and the whole tree, the fitting accuracies of bole, branches, foliage leaves and roots by NSUR were slightly inferior to those by NAP, where the results were different from existing theories. After carefully thought theories, we speculate that reasons for this contradiction include: (I) The nonlinear parameter estimation method had affected the final results by NSUR. We only used MSM to calculate parameters, so we inferred that if we used other advanced methods to obtain parameters, the accuracies of the models would be improved; (II) Because existing uncertainty of predictive errors in every optimal model by SOF, error cancellation occurred during adjustment when we used NAP to process compatibility. Based on these two aspects, it might appear that the fitting accuracies of NAP were slightly better than NSUR, but there were no statistically significant differences between these two methods.

In addition to NAP and NSUR, Tang et al. [40] proposed another compatible estimation method called nonlinear joint equation sets estimation. This method is combined with the advantages of NSUR and NAP, but compatible models constructed by this approach have more parameters and more complicated model forms. Through theoretical consideration and empirical evidence, NSUR was recommended as a more suitable approach to solve compatibility in general.

### Optimization basic model of carbon content

In this study, we compared model performances based on different variables, such as D, DH, D^2^H and D&H. The results indicated that models with two dimensional variables (DH, D^2^H and D&H) were always superior to those with a single variable (D). Moreover, we found that the most useful predictor variable combination was D&H (Table 2 and Fig 2). Actually, DH or D^2^H are both special forms of D&H combinations from mathematical aspect. In practice, D and H are two easily collected biophysical properties of individual trees and generally available in most forest inventories. Many species-specific equations for relating D&H to standing volume of wood or total biomass (carbon content) are commonly used for forest ecological studies [36–39]. So we conclude that it was better to use D&H as variables to establish carbon content models in this study.

Existing studies show that the selection of model forms always depends on subjective experience instead of objective comparisons of different models. However, in this study the performance of four basic models were compared in order to find the optimal form for simulating carbon content. Specially, the general model proposed by this study could be turned into other three function models (i.e. power, exponential and polynomial functions) by setting specific values for certain parameters. Using the Duncan test, we found that there were no significant differences among the results of the four basic models with D as a single variable. However, when we introduced H as another variable, exponential function showed a significant difference and the fitting accuracies were lower than those of other functions (S9, S10, S11 Tables). Meanwhile, we found that the optimal model was the general model for the whole tree and all components except for roots, whose best model form was a polynomial function (Table 2). But before setting a polynomial function as a basic model, the optimal maximum order must first be determined. Besides, the form of a power function was relatively simple and analogous to the Constant Allometric Ratio (CAR) model [47] often used to estimate forest biomass, but accuracy of power function was inferior to the general model in some results (Table 2). So, on balance, we did not think that these two types were optimal basic models in this study, at least for precision and practicality.

To further compare performance, we listed some models used in estimation for forest aboveground biomass from previous literature [11, 12, 37, 39, 48–53]. These models were used to fit the modelling data from this study (Table 6 presents only examples of aboveground carbon content. Most of models were logarithmic transformation of a power function and this transformation could be used to deal with the problem of heteroscedasticity). From the evaluation indices, we observed that accuracies of foreign models were still lower than those of the general model from Table 2. The new general model had a widespread applicability, noticeable flexibility, and could adapt to different situations through proper transformation. Some commonly used functions were actually special forms of the general model. So we recommend the use of new general model to estimate the carbon content of individual trees as the optimal basic model with D&H in this study.

**Table 6:** Results of fitting modelling data by some common models usually used in estimation for forest biomass from previous literatures.

No.	R^2^	Mean Residual	Residual Variance	Mean Square Error	Model
**Model 1**	0.9737	0.7302	94.7392	9.7608	ln(W_2_) = ln(p)+qln(D)
**Model 2**	0.9742	0.5552	83.3121	9.1444	ln(W_2_) = ln(p)+qln(D)+s(ln(D))^2^
**Model 3**	0.9756	0.4223	56.9311	7.5571	ln(W_2_) = ln(p)+qln(D)+s(ln(D))^2^+t(ln(D))^3^
**Model 4** ^a^	0.9737	0.7302	94.7392	9.7608	ln(W_2_) = ln(p)+qln(D)+sln(ρ)
**Model 5** ^b^	0.9742	0.5552	83.3121	9.1444	ln(W_2_) = ln(p)+qln(D+s(ln(D))^2^+tln(ρ)
**Model 6** ^c^	0.9756	0.4223	56.9311	7.5571	ln(W_2_) = ln(p)+qln(D)+s(ln(D))^2^+t(ln(D))^3^+fln(ρ)
**Model 7** ^c^	0.9756	0.4223	56.9311	7.5571	ln(W_2_) = ln(p)+qln(D)+s(ln(D))^2^+t(ln(D))^3^+ln(ρ)
**Model 8**	0.9908	0.1343	11.9312	3.4568	ln(W_2_) = ln(p)+qln(D)+sln(H)
**Model 9**	0.9908	0.1526	12.6990	3.5668	ln(W_2_) = ln(p)+qln(D^2^H)
**Model 10** ^d^	0.9908	0.1343	11.9312	3.4568	ln(W_2_) = ln(p)+qln(D)+sln(H)+tln(ρ)
**Model 11** ^e^	0.9908	0.1526	12.6990	3.5668	ln(W_2_) = ln(p)+qln(D^2^H)+tln(ρ)
**Model 12** ^e^	0.9908	0.1526	12.6990	3.5668	ln(W_2_) = ln(p)+qln(D^2^Hρ)
**Model 13**	0.9918	0.1427	11.6311	3.4134	W_2_ = W_3_+ W_4_+ W_5_
	0.9922	0.1258	8.6308	2.9405	W_3_ = p(D^2^H) ^q^
	0.9080	0.0296	0.5876	0.7671	W_4_ = p(W_3_) ^q^
	0.9254	-0.0127	0.3553	0.5962	W_5_ = p (W_3_+ W_4_) ^q^

D represented diameter at breast height; H represented tree height; W_2_, W_3_, W_4_, W_5_, W_6_ represented carbon content of aboveground, bole, branches, foliage leaves and roots, respectively; ρ represented wood specific gravity, ρ = 0.307 for Chinese fir (by “Afforestation Project Carbon Measurement and Monitoring Guidelines”, language in Chinese, State Forestry Administration, 2011), because ρ was constant in this study, some models results were same. In the models, p, q, s, t were parameters.

^a^ The results were same as model 1 because ρ was constant for Chinese fir in this study;

^b^ The results were same as model 2 because ρ was constant for Chinese fir in this study;

^c^ The results were same as model 3 because ρ was constant for Chinese fir in this study;

^d^ The results were same as model 8 because ρ was constant for Chinese fir in this study;

^e^ The results were same as model 9 because ρ was constant for Chinese fir in this study.

**Fig 3:**
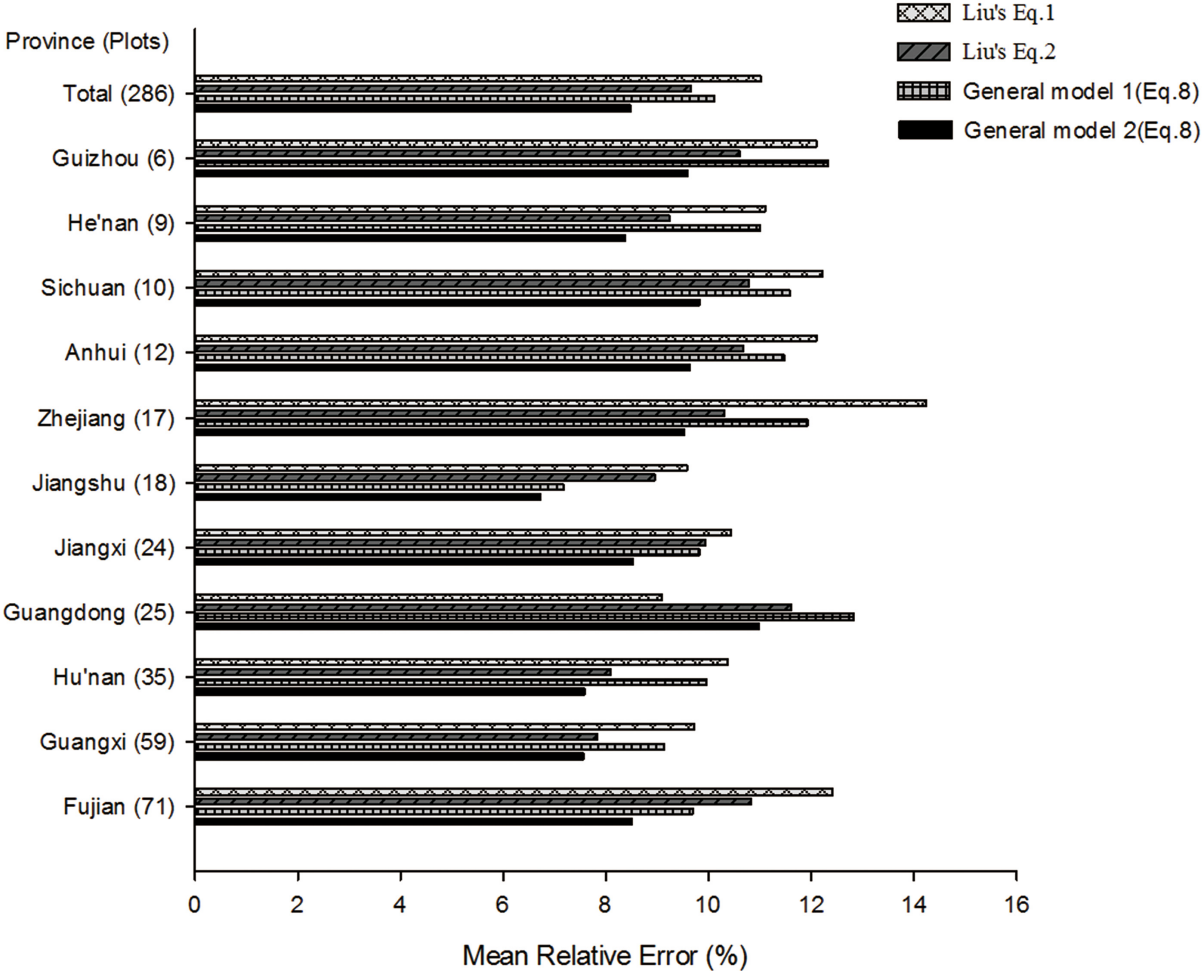
The comparisons of mean relative error of the whole tree carbon content predictions between Liu’s general model and the general model proposed by this study based on a regional database of individual trees of Chinese fir collected by Liu(2010).

For a more detailed examination of the general model extrapolation, we made comparisons between Liu’s general model and our general model based on a regional database of individual trees of Chinese fir collected by Liu [54]. We used model (Eq 8 form) from Table 4 by SOF for the whole tree as General model 1 and constructed new fitting model (Eq 8 form) by SOF for the whole tree based on Liu’ s regional database as General model 2. Fig 3 demonstrates the mean relative error of the whole tree carbon content prediction by using four models. The result revealed that, for total province plots, the General model 2 had the best performance on estimating the whole tree carbon content. The prediction ability of the General model 1 was superior to that of Liu’ Eq 1, but inferior to that of Liu’s Eq 2. However, the General model 1 had better results than that of Liu’s Eqs 1 and 2 for Fujian Province plots. So results from Fig 3 indicate that estimated errors were exposed and amplified when we used the General model 1 to fit regional database. The reason might be the General model 1 was constructed only based on plots of Fujian Province in this study. Over-fitting problems appeared when estimating other provinces’ plots due to existing variability in the real world. So it was not suitable to use directly the General model 1 to predict carbon content of Chinese fir in other provinces. Hence, a new fitting general model was constructed to provide further comparison. The results of the General model 2 revealed that the form of Eq 8 was optimal model for estimating carbon content in the mathematical significance for all provinces except for Guangdong, whose best results were based on Liu’ Eq 1. Therefore, we still recommend trying to use the general model (Eq 8) as an optimal form to estimate carbon content even based on meta-analysis of published data to date.

Liu’s Eq 1:ln *W* = *a*_1_+*b*_1_ ln *D*; Liu’s Eq 2: ln *W* = *a*_2_+*b*_2_ ln(*D*^2^*H*). *W* represented carbon content, *a*_1_, *a*_2_, *b*_1_, *b*_1_ were parameters. Because Liu’s database was about biomass for the whole tree, we used the general carbon content ratio of Chinese fir (0.52, by “Afforestation Project Carbon Measurement and Monitoring Guidelines”, language in Chinese, State Forestry Administration, 2011) to convert biomass to carbon content.

The General model 1 represented the general model (Eq 8 form) from Table 4 by SOF for the whole tree. The General model 2 represented the new fitting general model (Eq 8 form) by SOF for the whole tree based on Liu’s regional database.

The formula of relative error was $(\frac{\left|estimation-observation\right|}{observation})\times 100\%$

### Limitations and recommendations

We established different models of individual trees based on the perspective of carbon content. These models had excellent abilities and strong operability. However, errors in model extrapolation have two main sources in general [55]: the stochastic character of the estimated model coefficients and measurement errors in the data or lack of data used for model construction. Therefore there were still some aspects that should be given attention for practical application.

First, although the new general model had good performance, there were some issues to be paid attentions. Because this model required more parameters to be estimated and the initial setting values of parameters had an effect on the final results. Then the phenomenon of slow convergence might have appeared in the process of nonlinear parameter estimation. So we must fine tune the convergence criterion to obtain the desired degree of accuracy by trying different algorithms, such as a genetic algorithm or simulated annealing algorithm.

Second, in order to obtain more precise models, all results in this study were kept in four decimal places and most of the conclusions were drawn based on statistical significance. We didn’t rely entirely on statistical tests to decide optimal model in practice because differences in the third or fourth decimal place have no impacts at all in biological or ecological significance due to naturally occurring variability in the real world. Like results in S8, S9, S10 and S11 Tables, we found that there was no significant differences (at 0.01 significance level) among different model forms with different variables except between Eq 2 and other three models (Eqs 1, 3 and 7) with variable DH (S9 Table). Moreover, some results showed that the final form of the general model almost transformed into a power function (S10 and S11 Tables), i.e. carbon content might typically be characterized by an allometric scaling law in some extent [56]. So it is an alternative choice to use CAR model to estimate carbon content of individual trees of Chinese fir without extremely high precision demand in practice.

Third, we only used D and H to estimate carbon content of each component. We found that the estimated values of branches and foliage leaves had relatively larger deviations than other components. In some previous studies, scholars suggested that crown breath is a key variable to influence biomass of branches and foliage leaves [15, 40]. So if we can add crown breath as another variable to model, the results may be more accurate. Moreover, specific gravity is becoming another important variable for biomass estimation [57–58]. Although the whole tree specific gravity will not be a useful prediction variable in practice due to that it is seldom measured in stand survey, it still could be a very good variable for theoretical model guidance and selection when we develop model extrapolation for other species (like some models in Table 6).

Furthermore, most validation data from previous studies were only on biomass of Chinese fir, not carbon content. There were some deviations when we directly converted biomass into carbon content by using average carbon content ratios. However, different sampling and measurement methods might have led to inconsistence and inaccuracy during biomass data collection. Some models were extrapolated poorly in some extent, such as the estimate values of branches and foliage leaves which had relatively large deviations for validation data in Table 4. So increasing quantification of potential error would be useful for practical application in further studies.

## Conclusion

Accurate estimation of forest carbon content plays a major role in sustainable management of forests as well as mitigating the increasing CO_2_ concentration in the atmosphere. Based on the carbon content data on the Chinese fir plantation in Fujian Province, we selected four basic models to establish carbon content models. The results indicated that models with two dimensional variables (DH, D^2^H and D&H) were always superior to those with a single variable (D) and the most useful predictor variable combination was D&H. Through theoretical analysis and predictive validation based on data from previous studies, we recommend using NSUR to guarantee additivity when constructing a compatibility model. Meanwhile, comparisons of model evaluation indices showed that the general model was superior to other present models for estimating carbon content in statistical or mathematical significance. Theoretically, the general model has more flexibility and higher availability because it could transform to different form models by setting specific initial parameters. Hence, we conclude that the general model should be worthy of promotion for predicting carbon content of individual trees, not only for Chinese fir, but also for other vegetation types.

## Supporting Information

S1 Table The table of quadratic orthogonal rotational combining design. (DOCX)

S2 Table Details for modelling samples(carbon content). (DOCX)

S3 Table Details for validation samples (biomass). (DOCX)

S4 Table Weighted functions of four basic models of different components with variable D. (DOCX)

S5 Table Weighted functions of four basic models of different components with variable DH. (DOCX)

S6 Table Weighted functions of four basic models of different components with variable D^2^H. (DOCX)

S7 Table Weighted functions of four basic models of different components with variables D&H. (DOCX)

S8 Table Comparison evaluation indices of four basic models with variable D. (DOCX)

S9 Table Comparison evaluation indices of four basic models with variable DH. (DOCX)

S10 Table Comparison evaluation indices of four basic models with variable D^2^H. (DOCX)

S11 Table Comparison evaluation indices of four basic models with variables D&H. (DOCX)
