# Supplementary material for: Compatible Models of Carbon Content of Individual Trees on a Cunninghamia lanceolata Plantation in Fujian Province, China
Source: PLoS One. 2016 Mar 16;11(3):e0151527. doi: 10.1371/journal.pone.0151527 (PMC4794127; doi:10.1371/journal.pone.0151527)
Supplement: S1 Table — (DOCX) [file pone.0151527.s001.docx]

The table of quadratic orthogonal rotational combining design.

| Plot No. | Row 1 | Row 2 | Row 3 | Row 4 | Row 5 | Row 6 | Row 7 | Row 8 |
| --- | --- | --- | --- | --- | --- | --- | --- | --- |
|  | slope aspect | stand age | Stand density | site index | cultivation target | slope gradient | slope position | Blank |
| 1 | 1 | 1 | 1 | 1 | 1 | 1 | 1 | 1 |
| 2 | 1 | 1 | 2 | 2 | 2 | 2 | 2 | 2 |
| 3 | 1 | 1 | 3 | 3 | 3 | 3 | 3 | 3 |
| 4 | 1 | 2 | 1 | 1 | 2 | 2 | 3 | 3 |
| 5 | 1 | 2 | 2 | 2 | 3 | 3 | 1 | 1 |
| 6 | 1 | 2 | 3 | 3 | 1 | 1 | 2 | 2 |
| 7 | 1 | 3 | 1 | 2 | 1 | 3 | 2 | 3 |
| 8 | 1 | 3 | 2 | 3 | 2 | 1 | 3 | 1 |
| 9 | 1 | 3 | 3 | 1 | 3 | 2 | 1 | 2 |
| 10 | 2 | 1 | 1 | 3 | 3 | 2 | 2 | 1 |
| 11 | 2 | 1 | 2 | 1 | 1 | 3 | 3 | 2 |
| 12 | 2 | 1 | 3 | 2 | 2 | 1 | 1 | 3 |
| 13 | 2 | 2 | 1 | 2 | 3 | 1 | 3 | 2 |
| 14 | 2 | 2 | 2 | 3 | 1 | 2 | 1 | 3 |
| 15 | 2 | 2 | 3 | 1 | 2 | 3 | 2 | 1 |
| 16 | 2 | 3 | 1 | 3 | 2 | 3 | 1 | 2 |
| 17 | 2 | 3 | 2 | 1 | 3 | 1 | 2 | 3 |
| 18 | 2 | 3 | 3 | 2 | 1 | 2 | 3 | 1 |

Note: Slope aspect: 1 represented sunny slope, 2 represented shady slope; Stand age: 1 represented 6～12 years, 2 represented 12～18 years; 3 represented 18～26 years; Stand density: 1 represented 3000～4000 plants/hm^2^, 2 represented 2000～3000 plants/hm^2^; 3 represented 1000～2000 plants/hm^2^; Site index: 1 represented 6～12m, 2 represented 12～20m; 3 represented 20～28m; Cultivation target: : 1 represented small-diameter wood, 2 represented medium-diameter wood; 3 represented big-diameter wood; Slope gradient:1 represented flat slope, 2 represented gentle slope; 3 represented steep slope; Slope position: 1 represented lower slope, 2 represented middle slope; 3represented upper slope.
