# Supplementary material for: Compatible Models of Carbon Content of Individual Trees on a Cunninghamia lanceolata Plantation in Fujian Province, China
Source: PLoS One. 2016 Mar 16;11(3):e0151527. doi: 10.1371/journal.pone.0151527 (PMC4794127; doi:10.1371/journal.pone.0151527)
Supplement: S2 Table — (DOCX) [file pone.0151527.s002.docx]

Details for modelling samples(carbon content)

| No | D(cm) | H(m) | Volume(m3) | D^2^H | DH | Bole(kg) | Branches(kg) | Foliage leaves(kg) | Roots(kg) | Aboveground(kg) | Whole tree(kg) |
| --- | --- | --- | --- | --- | --- | --- | --- | --- | --- | --- | --- |
| 1 | 8.60 | 11.40 | 0.0344 | 843.1440 | 98.0400 | 6.3074 | 1.4170 | 0.7534 | 2.4167 | 8.4777 | 10.8944 |
| 2 | 8.90 | 12.20 | 0.0390 | 966.3620 | 108.5800 | 9.8847 | 1.5350 | 0.8360 | 3.2760 | 12.2557 | 15.5317 |
| 3 | 9.20 | 12.60 | 0.0429 | 1066.4640 | 115.9200 | 11.4495 | 1.5862 | 0.8327 | 2.8033 | 13.8684 | 16.6717 |
| 4 | 10.20 | 12.80 | 0.0532 | 1331.7120 | 130.5600 | 11.9715 | 1.9800 | 1.1115 | 2.9686 | 15.0630 | 18.0315 |
| 5 | 10.50 | 10.95 | 0.0490 | 1207.2375 | 114.9750 | 10.3209 | 1.9457 | 1.1499 | 3.3833 | 13.4165 | 16.7998 |
| 6 | 11.00 | 13.90 | 0.0664 | 1681.9000 | 152.9000 | 15.2434 | 2.1572 | 1.2490 | 4.4080 | 18.6496 | 23.0576 |
| 7 | 11.20 | 14.50 | 0.0714 | 1818.8800 | 162.4000 | 14.0635 | 2.1150 | 1.2298 | 4.5174 | 17.4082 | 21.9256 |
| 8 | 11.80 | 14.56 | 0.0794 | 2027.3344 | 171.8080 | 18.4305 | 2.7800 | 2.2088 | 5.1610 | 23.4193 | 28.5802 |
| 9 | 12.00 | 10.60 | 0.0618 | 1526.4000 | 127.2000 | 15.8825 | 2.7282 | 1.2986 | 4.7077 | 19.9092 | 24.6169 |
| 10 | 12.60 | 13.80 | 0.0860 | 2190.8880 | 173.8800 | 18.6397 | 4.3250 | 1.6435 | 5.0400 | 24.6082 | 29.6482 |
| 11 | 13.20 | 14.50 | 0.0985 | 2526.4800 | 191.4000 | 22.2589 | 2.8200 | 3.0495 | 6.5520 | 28.1284 | 34.6804 |
| 12 | 13.80 | 13.60 | 0.1014 | 2589.9840 | 187.6800 | 23.9325 | 2.9300 | 3.1493 | 5.8262 | 30.0117 | 35.8380 |
| 13 | 14.70 | 13.90 | 0.1170 | 3003.6510 | 204.3300 | 24.5235 | 2.9200 | 3.1018 | 10.5840 | 30.5452 | 41.1292 |
| 14 | 15.60 | 14.20 | 0.1340 | 3455.7120 | 221.5200 | 35.8255 | 2.8150 | 3.0638 | 9.0720 | 41.7043 | 50.7763 |
| 15 | 16.00 | 14.40 | 0.1425 | 3686.4000 | 230.4000 | 35.9667 | 2.2206 | 3.0254 | 11.5517 | 41.2128 | 52.7644 |
| 16 | 16.60 | 15.30 | 0.1617 | 4216.0680 | 253.9800 | 39.1758 | 3.8068 | 3.3506 | 8.7967 | 46.3332 | 55.1299 |
| 17 | 16.70 | 13.80 | 0.1492 | 3848.6820 | 230.4600 | 40.6110 | 2.7800 | 3.3060 | 11.5920 | 46.6970 | 58.2890 |
| 18 | 17.20 | 14.80 | 0.1683 | 4378.4320 | 254.5600 | 40.5848 | 5.0115 | 4.6835 | 16.6320 | 50.2798 | 66.9118 |
| 19 | 17.60 | 15.60 | 0.1845 | 4832.2560 | 274.5600 | 42.4258 | 6.5244 | 6.0667 | 10.0534 | 55.0168 | 65.0702 |
| 20 | 18.40 | 14.60 | 0.1897 | 4942.9760 | 268.6400 | 39.7742 | 4.9107 | 4.5144 | 10.2467 | 49.1992 | 59.4459 |
| 21 | 21.00 | 14.40 | 0.2426 | 6350.4000 | 302.4000 | 53.6441 | 6.5561 | 2.9620 | 13.9200 | 63.1622 | 77.0822 |
| 22 | 23.00 | 14.30 | 0.2880 | 7564.7000 | 328.9000 | 57.0436 | 4.6527 | 3.6678 | 20.0584 | 65.3641 | 85.4225 |
| 23 | 24.90 | 14.92 | 0.3494 | 9250.5492 | 371.5080 | 68.6709 | 6.1545 | 5.4000 | 21.1701 | 80.2254 | 101.3955 |
| 24 | 25.50 | 18.20 | 0.4372 | 11834.5500 | 464.1000 | 103.4128 | 5.7630 | 7.4307 | 23.3934 | 116.6065 | 139.9999 |
| 25 | 27.40 | 21.40 | 0.5816 | 16066.2640 | 586.3600 | 143.8124 | 12.4777 | 9.4173 | 27.5501 | 165.7075 | 193.2575 |
| 26 | 28.70 | 22.50 | 0.6659 | 18533.0250 | 645.7500 | 153.9294 | 12.1182 | 9.0802 | 29.3094 | 175.1278 | 204.4372 |
| 27 | 8.40 | 11.46 | 0.0330 | 808.6176 | 96.2640 | 7.9758 | 1.9300 | 1.4868 | 4.4201 | 11.3925 | 15.8126 |
| 28 | 9.80 | 12.16 | 0.0470 | 1167.8464 | 119.1680 | 10.8104 | 2.1750 | 1.6435 | 4.7275 | 14.6289 | 19.3564 |
| 29 | 10.90 | 13.50 | 0.0635 | 1603.9350 | 147.1500 | 15.6011 | 2.4700 | 1.9475 | 5.6902 | 20.0186 | 25.7088 |
| 30 | 12.80 | 13.48 | 0.0869 | 2208.5632 | 172.5440 | 19.9315 | 2.7200 | 2.3228 | 6.5470 | 24.9743 | 31.5212 |
| 31 | 12.50 | 14.41 | 0.0880 | 2251.5625 | 180.1250 | 20.2401 | 2.7550 | 2.2848 | 6.8342 | 25.2799 | 32.1141 |
| 32 | 13.50 | 13.55 | 0.0968 | 2469.4875 | 182.9250 | 21.5110 | 3.0500 | 2.3608 | 7.2626 | 26.9217 | 34.1844 |
| 33 | 14.20 | 14.37 | 0.1127 | 2897.5668 | 204.0540 | 26.4847 | 3.2550 | 2.8358 | 7.3181 | 32.5755 | 39.8936 |
| 34 | 12.50 | 13.82 | 0.0848 | 2159.3750 | 172.7500 | 18.6188 | 2.8850 | 2.3513 | 6.4663 | 23.8551 | 30.3214 |
| 35 | 11.30 | 13.14 | 0.0665 | 1677.8466 | 148.4820 | 14.9630 | 2.4750 | 2.0853 | 5.5339 | 19.5233 | 25.0572 |
| 36 | 14.90 | 14.65 | 0.1259 | 3252.4465 | 218.2850 | 29.1154 | 3.3000 | 3.0210 | 7.9078 | 35.4364 | 43.3442 |
| 37 | 14.20 | 14.81 | 0.1157 | 2986.2884 | 210.3020 | 25.1563 | 3.2600 | 2.8310 | 7.9430 | 31.2473 | 39.1903 |
| 38 | 16.30 | 14.82 | 0.1517 | 3937.5258 | 241.5660 | 35.5483 | 3.8500 | 3.3345 | 9.3694 | 42.7328 | 52.1022 |
| 39 | 16.50 | 14.17 | 0.1492 | 3857.7825 | 233.8050 | 34.9469 | 3.7650 | 3.4010 | 9.2786 | 42.1129 | 51.3915 |
| 40 | 16.70 | 13.75 | 0.1487 | 3834.7375 | 229.6250 | 32.9542 | 3.8750 | 3.2870 | 8.9964 | 40.1162 | 49.1126 |
| 41 | 18.90 | 14.73 | 0.2015 | 5261.7033 | 278.3970 | 46.7196 | 4.6150 | 4.1563 | 11.3803 | 55.4908 | 66.8712 |
| 42 | 17.10 | 14.31 | 0.1614 | 4184.3871 | 244.7010 | 36.5577 | 4.0650 | 3.5720 | 10.0397 | 44.1947 | 54.2344 |
| 43 | 20.60 | 13.78 | 0.2246 | 5847.6808 | 283.8680 | 52.4883 | 5.1850 | 4.3368 | 12.3782 | 62.0100 | 74.3883 |
| 44 | 20.20 | 13.89 | 0.2177 | 5667.6756 | 280.5780 | 47.0334 | 5.1350 | 4.2798 | 12.7764 | 56.4481 | 69.2245 |
| 45 | 19.10 | 14.28 | 0.2000 | 5209.4868 | 272.7480 | 46.4163 | 4.7500 | 4.0423 | 11.3047 | 55.2085 | 66.5132 |
| 46 | 22.10 | 14.66 | 0.2724 | 7160.0906 | 323.9860 | 62.3887 | 5.8200 | 5.1823 | 14.6916 | 73.3909 | 88.0825 |
| 47 | 22.40 | 15.04 | 0.2861 | 7546.4704 | 336.8960 | 66.6041 | 6.1900 | 5.3580 | 15.0696 | 78.1521 | 93.2217 |
| 48 | 24.60 | 17.01 | 0.3836 | 10293.7716 | 418.4460 | 83.7218 | 8.3450 | 6.9018 | 20.0844 | 98.9686 | 119.0530 |
| 49 | 24.10 | 16.27 | 0.3542 | 9449.7787 | 392.1070 | 81.7815 | 7.7800 | 6.3983 | 18.6178 | 95.9598 | 114.5775 |
| 50 | 26.80 | 18.89 | 0.4982 | 13567.5536 | 506.2520 | 119.7252 | 9.9950 | 8.9775 | 24.1567 | 138.6977 | 162.8544 |
| 51 | 25.70 | 17.76 | 0.4343 | 11730.3024 | 456.4320 | 99.6786 | 9.1300 | 8.0370 | 23.0026 | 116.8456 | 139.8481 |
| 52 | 13.00 | 12.60 | 0.0843 | 2129.4000 | 163.8000 | 20.3290 | 2.8050 | 2.2325 | 6.4764 | 25.3665 | 31.8429 |
| 53 | 14.00 | 12.50 | 0.0967 | 2450.0000 | 175.0000 | 23.1689 | 2.8450 | 2.3418 | 7.1971 | 28.3557 | 35.5528 |
| 54 | 19.00 | 22.50 | 0.2973 | 8122.5000 | 427.5000 | 67.7390 | 6.5050 | 5.4910 | 16.5715 | 79.7350 | 96.3065 |

The roots included large sized roots (2-5 cm), middle sized roots (0.5-2 cm), small sized roots (0.2-0.5 cm), fine roots (< 0.2 cm) and stump roots (> 5cm).
