# Supplementary material for: Compatible Models of Carbon Content of Individual Trees on a Cunninghamia lanceolata Plantation in Fujian Province, China
Source: PLoS One. 2016 Mar 16;11(3):e0151527. doi: 10.1371/journal.pone.0151527 (PMC4794127; doi:10.1371/journal.pone.0151527)
Supplement: S3 Table — (DOCX) [file pone.0151527.s003.docx]

Details for validation samples (biomass).

| No | D(cm) | H(m) | Bole(kg) | Branches(kg) | Foliage leaves(kg) | Roots(kg) | Aboveground (kg) | Whole tree(kg) |
| --- | --- | --- | --- | --- | --- | --- | --- | --- |
| 1 | 5.00 | 6.22 | 2.51 | 1.83 | 0.82 | 1.27 | 5.16 | 6.43 |
| 2 | 8.50 | 7.10 | 10.49 | 3.91 | 3.70 | 4.44 | 18.10 | 22.54 |
| 3 | 6.17 | 8.76 | 5.39 | 2.41 | 0.79 | 2.99 | 8.60 | 11.59 |
| 4 | 15.24 | 14.19 | 66.41 | 7.96 | 7.44 | 15.82 | 81.81 | 97.63 |
| 5 | 15.44 | 14.97 | 66.12 | 8.10 | 7.56 | 17.33 | 81.78 | 99.11 |
| 6 | 14.68 | 15.11 | 53.43 | 6.83 | 7.12 | 15.80 | 67.38 | 83.18 |
| 7 | 21.00 | 17.99 | 101.80 | 12.09 | 8.87 | 25.11 | 122.76 | 147.87 |
| 8 | 19.39 | 18.72 | 115.36 | 14.64 | 10.29 | 30.26 | 140.29 | 170.55 |
| 9 | 19.83 | 19.45 | 130.03 | 11.27 | 11.94 | 36.46 | 153.24 | 189.70 |
| 10 | 20.24 | 20.19 | 145.93 | 11.57 | 13.86 | 43.92 | 171.36 | 215.28 |
| 11 | 20.61 | 20.92 | 162.95 | 11.85 | 16.08 | 33.41 | 190.88 | 224.30 |
| 12 | 11.70 | 8.63 | 19.57 | 5.26 | 4.61 | 7.02 | 29.44 | 36.46 |
| 13 | 14.32 | 14.41 | 54.48 | 7.12 | 6.91 | 12.08 | 68.51 | 80.59 |
| 14 | 16.92 | 15.46 | 66.17 | 6.91 | 5.93 | 22.25 | 79.01 | 101.26 |
| 15 | 12.50 | 9.60 | 23.44 | 6.50 | 4.45 | 11.43 | 34.39 | 45.82 |
| 16 | 12.90 | 9.80 | 24.91 | 6.82 | 4.53 | 11.75 | 36.26 | 48.01 |
| 17 | 13.30 | 10.20 | 27.32 | 6.61 | 4.66 | 11.93 | 38.59 | 50.52 |
| 18 | 19.31 | 18.19 | 98.19 | 9.72 | 9.88 | 22.80 | 117.79 | 140.59 |
| 19 | 20.18 | 22.10 | 140.07 | 11.53 | 14.21 | 31.83 | 165.81 | 197.64 |
| 20 | 8.11 | 6.25 | 6.37 | 3.46 | 3.11 | 3.89 | 12.94 | 16.83 |
| 21 | 6.00 | 6.69 | 3.93 | 2.33 | 2.44 | 1.94 | 8.70 | 10.64 |
| 22 | 7.00 | 7.70 | 5.83 | 2.85 | 2.93 | 2.77 | 11.62 | 14.39 |
| 23 | 8.00 | 8.43 | 8.10 | 3.40 | 3.44 | 3.77 | 14.94 | 18.72 |
| 24 | 9.00 | 9.17 | 10.94 | 3.97 | 3.96 | 4.95 | 18.88 | 23.83 |
| 25 | 19.00 | 16.52 | 77.80 | 8.24 | 9.69 | 27.70 | 95.73 | 123.43 |
| 26 | 20.00 | 17.25 | 89.26 | 9.98 | 10.14 | 31.18 | 109.38 | 140.56 |
| 27 | 18.16 | 17.51 | 91.88 | 10.03 | 9.18 | 24.96 | 111.09 | 136.05 |

The bole included bark and the roots included large sized roots (2-5 cm), middle sized roots (0.5-2 cm), small sized roots (0.2-0.5 cm), fine roots (< 0.2 cm) and stump roots (> 5cm).
