# Supplementary material for: Compatible Models of Carbon Content of Individual Trees on a Cunninghamia lanceolata Plantation in Fujian Province, China
Source: PLoS One. 2016 Mar 16;11(3):e0151527. doi: 10.1371/journal.pone.0151527 (PMC4794127; doi:10.1371/journal.pone.0151527)
Supplement: S4 Table — (DOCX) [file pone.0151527.s004.docx]

Weighted functions of four basic models of different components with variable D

| Basic model | Bole | Branches | Foliage leaves | Roots | Aboveground | Whole tree |
| --- | --- | --- | --- | --- | --- | --- |
| Eq. 1 | $g(D)=D^{4.05}$ | $g(D)=D^{3.78}$ | $g(D)=D^{1.12}$ | $g(D)=D^{2.07}$ | $g(D)=D^{3.39}$ | $g(D)=D^{3.46}$ |
| Eq. 2 | $g(D)=D^{2.54}$ | $g(D)=D^{2.71}$ | $g(D)=D^{0.61}$ | $g(D)=D^{0.77}$ | $g(D)=D^{2.31}$ |  |
| Eq. 3 | $g(D)=D^{3.91}$ | $g(D)=D^{3.02}$ | $g(D)=D^{0.93}$ | $g(D)=D^{1.49}$ | $g(D)=D^{3.35}$ | $g(D)=D^{2.78}$ |
| Eq. 7 | $g(D)=D^{2.99}$ | $g(D)=D^{2.50}$ | $g(D)=D^{0.91}$ | $g(D)=D^{1.29}$ | $g(D)=D^{3.32}$ | $g(D)=D^{2.99}$ |

Note: Eq.1, Eq. 2, Eq. 3, Eq. 7 represented power, exponential, polynomial functions and the general model, respectively.
