# Supplementary material for: Compatible Models of Carbon Content of Individual Trees on a Cunninghamia lanceolata Plantation in Fujian Province, China
Source: PLoS One. 2016 Mar 16;11(3):e0151527. doi: 10.1371/journal.pone.0151527 (PMC4794127; doi:10.1371/journal.pone.0151527)
Supplement: S5 Table — (DOCX) [file pone.0151527.s005.docx]

Weighted functions of four basic models of different components with variable DH.

| Basic model | Bole | Branches | Foliage leaves | Roots | Aboveground | Whole tree |
| --- | --- | --- | --- | --- | --- | --- |
| Eq. 1 | $g(DH)=(DH)^{1.35}$ | $g(DH)=(DH)^{2.47}$ | $g(DH)=(DH)^{1.25}$ | $g(DH)=(DH)^{0.45}$ | $g(DH)=(DH)^{2.05}$ | $g(DH)=(DH)^{1.40}$ |
| Eq. 2 | $g(DH)=(DH)^{-0.33}$ | $g(DH)=(DH)^{1.17}$ | $g(DH)=(DH)^{0.27}$ | $g(DH)=(DH)^{0.12}$ | $g(DH)=(DH)^{-0.15}$ | $g(DH)=(DH)^{-0.41}$ |
| Eq. 3 | $g(DH)=(DH)^{1.78}$ | $g(DH)=(DH)^{2.58}$ | $g(DH)=(DH)^{1.56}$ | $g(DH)=(DH)^{0.07}$ | $g(DH)=(DH)^{1.58}$ | $g(DH)=(DH)^{1.10}$ |
| Eq. 7 | $g(DH)=(DH)^{1.35}$ | $g(DH)=(DH)^{2.60}$ | $g(DH)=(DH)^{0.76}$ | $g(DH)=(DH)^{0.42}$ | $g(DH)=(DH)^{2.05}$ | $g(DH)=(DH)^{0.95}$ |

Note: Eq.1, Eq. 2, Eq. 3, Eq. 7 represented power, exponential, polynomial functions and the general model, respectively.
