# Supplementary material for: Compatible Models of Carbon Content of Individual Trees on a Cunninghamia lanceolata Plantation in Fujian Province, China
Source: PLoS One. 2016 Mar 16;11(3):e0151527. doi: 10.1371/journal.pone.0151527 (PMC4794127; doi:10.1371/journal.pone.0151527)
Supplement: S6 Table — (DOCX) [file pone.0151527.s006.docx]

Weighted functions of four basic models of different components with variable D^2^H.

| Basic model | Bole | Branches | Foliage leaves | Roots | Aboveground | Whole |
| --- | --- | --- | --- | --- | --- | --- |
| Eq. 1 | $g(D^{2}H)=(D^{2}H)^{1.38}$ | $g(D^{2}H)=(D^{2}H)^{1.41}$ | $g(D^{2}H)=(D^{2}H)^{0.09}$ | $g(D^{2}H)=(D^{2}H)^{0.19}$ | $g(D^{2}H)=(D^{2}H)^{0.92}$ | $g(D^{2}H)=(D^{2}H)^{0.78}$ |
| Eq. 2 | $g(D^{2}H)=(D^{2}H)^{-0.13}$ | $g(D^{2}H)=(D^{2}H)^{0.28}$ | $g(D^{2}H)=(D^{2}H)^{-0.09}$ | $g(D^{2}H)=(D^{2}H)^{-0.26}$ | $g(D^{2}H)=(D^{2}H)^{-0.11}$ | $g(D^{2}H)=(D^{2}H)^{-0.11}$ |
| Eq. 3 | $g(D^{2}H)=(D^{2}H)^{1.33}$ | $g(D^{2}H)=(D^{2}H)^{0.95}$ | $g(D^{2}H)=(D^{2}H)^{-0.21}$ | $g(D^{2}H)=(D^{2}H)^{0.08}$ | $g(D^{2}H)=(D^{2}H)^{1.50}$ | $g(D^{2}H)=(D^{2}H)^{0.98}$ |
| Eq. 7 | $g(D^{2}H)=(D^{2}H)^{1.38}$ | $g(D^{2}H)=(D^{2}H)^{1.06}$ | $g(D^{2}H)=(D^{2}H)^{0.09}$ | $g(D^{2}H)=(D^{2}H)^{0.19}$ | $g(D^{2}H)=(D^{2}H)^{0.92}$ | $g(D^{2}H)=(D^{2}H)^{0.78}$ |

Note: Eq.1, Eq. 2, Eq. 3, Eq. 7 represented power, exponential, polynomial functions and the general model, respectively.
