# Supplementary material for: Compatible Models of Carbon Content of Individual Trees on a Cunninghamia lanceolata Plantation in Fujian Province, China
Source: PLoS One. 2016 Mar 16;11(3):e0151527. doi: 10.1371/journal.pone.0151527 (PMC4794127; doi:10.1371/journal.pone.0151527)
Supplement: S7 Table — (DOCX) [file pone.0151527.s007.docx]

Weighted functions of four basic models of different components with variables D & H.

| Basic model | Bole | Branches | Foliage leaves | Roots | Aboveground | Whole tree |
| --- | --- | --- | --- | --- | --- | --- |
| Eq. 4 | $g(D,H)=D^{3.02}H^{-1.17}$ | $g(D,H)=D^{1.77}H^{0.72}$ | $g(D,H)=D^{0.18}H^{0.06}$ | $g(D,H)=D^{1.61}H^{-2.62}$ | $g(D,H)=D^{4.40}H^{0.60}$ | $g(D,H)=D^{4.46}H^{0.41}$ |
| Eq. 5 | $g(D,H)=D^{4.39}H^{-1.52}$ | $g(D,H)=D^{1.74}H^{0.89}$ | $g(D,H)=D^{0.13}H^{-0.04}$ | $g(D,H)=D^{1.39}H^{-1.13}$ | $g(D,H)=D^{3.42}H^{0.76}$ | $g(D,H)=D^{2.11}H^{1.66}$ |
| Eq. 6 | $g(D,H)=D^{2.54}H^{-1.73}$ | $g(D,H)=D^{1.36}H^{0.63}$ | $g(D,H)=D^{0.73}H^{-0.38}$ | $g(D,H)=D^{0.18}H^{0.06}$ | $g(D,H)=D^{2.19}H^{0.26}$ | $g(D,H)=D^{2.43}H^{0.81}$ |
| Eq. 8 | $g(D,H)=D^{3.02}H^{-1.18}$ | $g(D,H)=D^{2.07}H^{0.30}$ | $g(D,H)=D^{0.18}H^{0.36}$ | $g(D,H)=D^{1.78}H^{-2.79}$ | $g(D,H)=D^{4.43}H^{0.40}$ | $g(D,H)=D^{4.78}H^{0.71}$ |

Note: Eq.4, Eq. 5, Eq. 6, Eq. 8 represented power, exponential, polynomial functions and the general model, respectively.
