# Supplementary material for: Compatible Models of Carbon Content of Individual Trees on a Cunninghamia lanceolata Plantation in Fujian Province, China
Source: PLoS One. 2016 Mar 16;11(3):e0151527. doi: 10.1371/journal.pone.0151527 (PMC4794127; doi:10.1371/journal.pone.0151527)
Supplement: S8 Table — (DOCX) [file pone.0151527.s008.docx]

Comparison evaluation indices of four basic models with variable D.

| Component | Model | R^2^ | Mean Residual | Residual Variance | Mean Square Error |
| --- | --- | --- | --- | --- | --- |
| Bole | Eq. 1 | 0.9469 | 0.7477 | 59.0815 | 7.7227 |
|  | Eq. 2 | 0.9627 | 0.0031 | 41.5139 | 6.4431 |
|  | Eq. 3 | 0.9481 | -5.56E-06 | 57.7881 | 7.6018 |
|  | Eq. 7^*^ | 0.9705 | -0.1155 | 32.8280 | 5.7307 |
| Branches | Eq. 1 | 0.8544 | 0.0445 | 0.9294 | 0.9651 |
|  | Eq. 2 | 0.8787 | 0.0094 | 0.7744 | 0.8801 |
|  | Eq. 3 | 0.8700 | -5.55E-06 | 0.8296 | 0.9108 |
|  | Eq. 7* | 0.8916 | -0.0017 | 0.6919 | 0.8318 |
| Foliage leaves | Eq. 1 | 0.8855 | 0.0063 | 0.5456 | 0.7387 |
|  | Eq. 2 | 0.8830 | -0.0179 | 0.5578 | 0.7470 |
|  | Eq. 3 | 0.8863 | -3.70E-06 | 0.5416 | 0.7359 |
|  | Eq. 7* | 0.8938 | -0.0105 | 0.5060 | 0.7114 |
| Roots | Eq. 1 | 0.9471 | 0.0408 | 2.3694 | 1.5398 |
|  | Eq. 2 | 0.9446 | -0.0627 | 2.4814 | 1.5765 |
|  | Eq. 3 | 0.9484 | 1.85E-06 | 2.3097 | 1.5198 |
|  | Eq. 7* | 0.9517 | -0.0095 | 2.1635 | 1.4709 |
| Aboveground | Eq. 1 | 0.9450 | 0.8039 | 78.9807 | 8.9234 |
|  | Eq. 2 | 0.9612 | 0.0087 | 55.6996 | 7.4632 |
|  | Eq. 3 | 0.9476 | -3.70E-06 | 75.1300 | 8.6678 |
|  | Eq. 7* | 0.9732 | -0.1557 | 38.4567 | 6.2033 |
| Whole teee | Eq. 1 | 0.9526 | 0.8165 | 93.2356 | 9.6903 |
|  | Eq. 2 | 0.9664 | -0.0729 | 66.0521 | 8.1276 |
|  | Eq. 3 | 0.9556 | -5.55E-16 | 87.4257 | 9.3502 |
|  | Eq. 7* | 0.9771 | -0.1597 | 45.1548 | 6.7216 |

* represented the best basic model for estimating when using D as variable. Eq.1, Eq. 2, Eq. 3, Eq. 7 represented power, exponential, polynomial functions and the general model, respectively. Through Duncan’s multiple range tests, there were no significant differences (at 0.01 significant level) among different models.
