# Supplementary material for: Compatible Models of Carbon Content of Individual Trees on a Cunninghamia lanceolata Plantation in Fujian Province, China
Source: PLoS One. 2016 Mar 16;11(3):e0151527. doi: 10.1371/journal.pone.0151527 (PMC4794127; doi:10.1371/journal.pone.0151527)
Supplement: S9 Table — (DOCX) [file pone.0151527.s009.docx]

Comparison evaluation indices of four basic models with variable DH.

| Component | Model ^a^ | R^2^ | Mean Residual | Residual Variance | Mean Square Error |
| --- | --- | --- | --- | --- | --- |
| Bole | Eq. 1 | 0.9837 | -0.3136 | 18.0937 | 4.2652 |
|  | Eq. 2 | 0.9200 | -1.3637 | 89.0339 | 9.5338 |
|  | Eq. 3* | 0.9849 | -0.0008 | 16.8416 | 4.1038 |
|  | Eq. 7 | 0.9837 | -0.3135 | 18.0935 | 4.2652 |
| Branches | Eq. 1 | 0.9120 | 0.0105 | 0.5616 | 0.7495 |
|  | Eq. 2 | 0.8730 | -0.0516 | 0.8109 | 0.9020 |
|  | Eq. 3* | 0.9127 | -0.0008 | 0.5573 | 0.7465 |
|  | Eq. 7 | 0.9125 | 0.0023 | 0.5583 | 0.7472 |
| Foliage leaves | Eq. 1 | 0.9206 | -0.0291 | 0.3786 | 0.6160 |
|  | Eq. 2 | 0.8277 | -0.0694 | 0.8212 | 0.9089 |
|  | Eq. 3 | 0.9300 | 0.0008 | 0.3338 | 0.5778 |
|  | Eq. 7* | 0.9308 | 0.0001 | 0.3298 | 0.5743 |
| Roots | Eq. 1 | 0.9316 | -0.0932 | 3.0641 | 1.7529 |
|  | Eq. 2 | 0.8380 | -0.2237 | 7.2530 | 2.7024 |
|  | Eq. 3* | 0.9408 | 0.0008 | 2.6514 | 1.6283 |
|  | Eq. 7 | 0.9316 | -0.0932 | 3.0641 | 1.7529 |
| Aboveground | Eq. 1 | 0.9846 | -0.2984 | 22.0948 | 4.7100 |
|  | Eq. 2 | 0.9210 | -1.4541 | 113.3834 | 10.7470 |
|  | Eq. 3* | 0.9854 | -0.0008 | 20.9060 | 4.5723 |
|  | Eq. 7 | 0.9846 | -0.2982 | 22.0948 | 4.7100 |
| Whole tree | Eq. 1 | 0.9843 | -0.3947 | 30.9915 | 5.5810 |
|  | Eq. 2 | 0.9162 | -1.6680 | 164.9418 | 12.9508 |
|  | Eq. 3* | 0.9856 | -0.0008 | 28.3301 | 5.3226 |
|  | Eq. 7 | 0.9805 | -0.8406 | 38.3294 | 6.2479 |

* represented the best basic model for estimating when using DH as variable. Eq.1, Eq. 2, Eq. 3, Eq. 7 represented power, exponential, polynomial functions and the general model, respectively.

^a^ represented that through Duncan’s multiple range test, there was a significant difference (at 0.01 significant level) between Eq. 2 and other three models (Eq.1, Eq. 3, Eq. 7)
