# Supplementary material for: Compatible Models of Carbon Content of Individual Trees on a Cunninghamia lanceolata Plantation in Fujian Province, China
Source: PLoS One. 2016 Mar 16;11(3):e0151527. doi: 10.1371/journal.pone.0151527 (PMC4794127; doi:10.1371/journal.pone.0151527)
Supplement: S10 Table — (DOCX) [file pone.0151527.s010.docx]

Comparison evaluation indices of four basic models with variable D^2^H.

| Component | Model ^a^ | R^2^ | Mean Residual | Residual Variance | Mean Square Error |
| --- | --- | --- | --- | --- | --- |
| Bole ^b^ | Eq. 1* | 0.9922 | 0.1258 | 8.6308 | 2.9405 |
|  | Eq. 2 | 0.8970 | -1.4765 | 114.6798 | 10.8102 |
|  | Eq. 3 | 0.9920 | -0.3772 | 8.9058 | 3.0080 |
|  | Eq. 7 | 0.9922 | 0.1258 | 8.6308 | 2.9405 |
| Branches | Eq. 1 | 0.9080 | 0.0296 | 0.5876 | 0.7671 |
|  | Eq. 2 | 0.8480 | -0.0590 | 0.9703 | 0.9868 |
|  | Eq. 3 | 0.9142 | -7.41E-06 | 0.5477 | 0.7401 |
|  | Eq. 7* | 0.9155 | 0.0001 | 0.5391 | 0.7342 |
| Foliage leaves ^b^ | Eq. 1* | 0.9256 | -0.0121 | 0.3545 | 0.5955 |
|  | Eq. 2 | 0.7888 | -0.0702 | 1.0065 | 1.0057 |
|  | Eq. 3 | 0.9246 | 0.0000 | 0.3592 | 0.5993 |
|  | Eq. 7 | 0.9256 | -0.0121 | 0.3545 | 0.5955 |
| Roots ^b^ | Eq. 1* | 0.9559 | -0.0388 | 1.9724 | 1.4050 |
|  | Eq. 2 | 0.8118 | -0.2354 | 8.4276 | 2.9126 |
|  | Eq. 3 | 0.9585 | -3.70E-06 | 1.8563 | 1.3625 |
|  | Eq. 7 | 0.9559 | -0.0388 | 1.9724 | 1.4050 |
| Aboveground | Eq. 1 | 0.9917 | 0.1758 | 11.8663 | 3.4492 |
|  | Eq. 2 | 0.8968 | -1.5757 | 148.0862 | 12.2707 |
|  | Eq. 3* | 0.9921 | -9.26E-06 | 11.3360 | 3.3669 |
|  | Eq. 7 | 0.9917 | 0.1759 | 11.8663 | 3.4492 |
| Whole tree ^b^ | Eq. 1* | 0.9939 | 0.1256 | 11.9725 | 3.4624 |
|  | Eq. 2 | 0.8916 | -1.7979 | 213.3019 | 14.7151 |
|  | Eq. 3 | 0.9936 | 0.3772 | 12.5387 | 3.5610 |
|  | Eq. 7 | 0.9939 | 0.1256 | 11.9725 | 3.4624 |

^*^ represented the best basic model for estimating when using D^2^H as variable. Eq.1, Eq. 2, Eq. 3, Eq. 7 represented power, exponential, polynomial functions and the general model, respectively.

^a^ represented that through Duncan’s multiple range test, there was a significant difference (at 0.01 significant level) between Eq. 2 and other three models (Eq.1, Eq. 3, Eq. 7).

^b^ meant that under the condition of rounding four decimal places, results were almost the same between a power function (Eq.1) and the general model (Eq.7) for bole, foliage leaves, roots and the whole tree models. These showed that the final form of general models almost transformed into power function after calculating parameters. So considering Eq.1 had less parameters, we assumed Eq.1 was optimal due to more convenient application in practice.
