# Supplementary material for: Compatible Models of Carbon Content of Individual Trees on a Cunninghamia lanceolata Plantation in Fujian Province, China
Source: PLoS One. 2016 Mar 16;11(3):e0151527. doi: 10.1371/journal.pone.0151527 (PMC4794127; doi:10.1371/journal.pone.0151527)
Supplement: S11 Table — (DOCX) [file pone.0151527.s011.docx]

Comparison evaluation indices of four basic models with variable D & H.

| Component | Model ^a^ | R^2^ | Mean Residual | Residual Variance | Mean Square Error |
| --- | --- | --- | --- | --- | --- |
| Bole ^b^ | Eq. 4 | 0.9929 | 0.0314 | 7.9527 | 2.8202 |
|  | Eq. 5 | 0.9200 | -1.3637 | 89.0339 | 9.5338 |
|  | Eq. 6 | 0.9873 | 0.2247 | 14.0934 | 3.7608 |
|  | Eq. 8* | 0.9929 | -0.0958 | 7.9379 | 2.8191 |
| Branches | Eq. 4 | 0.9135 | 0.0173 | 0.5519 | 0.7431 |
|  | Eq. 5 | 0.8730 | -0.0516 | 0.8109 | 0.9020 |
|  | Eq. 6 | 0.9099 | 0.0106 | 0.5750 | 0.7584 |
|  | Eq. 8* | 0.9167 | 0.0003 | 0.5320 | 0.7294 |
| Foliage leaves | Eq. 4 | 0.9268 | -0.0171 | 0.3491 | 0.5911 |
|  | Eq. 5 | 0.8277 | -0.0694 | 0.8212 | 0.9089 |
|  | Eq. 6 | 0.9238 | -0.0081 | 0.3630 | 0.6026 |
|  | Eq. 8* | 0.9315 | -1.6667E-05 | 0.3264 | 0.5713 |
| Roots | Eq. 4 | 0.9592 | -0.0088 | 1.8247 | 1.3509 |
|  | Eq. 5 | 0.8380 | -0.2237 | 7.2530 | 2.7024 |
|  | Eq. 6* | 0.9598 | -0.0028 | 1.7999 | 1.3416 |
|  | Eq. 8 | 0.9593 | 0.0004 | 1.8204 | 1.3492 |
| Aboveground | Eq. 4 | 0.9926 | 0.0522 | 10.5416 | 3.2472 |
|  | Eq. 5 | 0.9210 | -1.4541 | 113.3834 | 10.7470 |
|  | Eq. 6 | 0.9874 | 0.2192 | 18.0159 | 4.2502 |
|  | Eq. 8* | 0.9928 | -0.0128 | 10.45408 | 3.2333 |
| Whole tree | Eq. 4 | 0.9942 | 0.0408 | 11.2791 | 3.3587 |
|  | Eq. 5 | 0.9162 | -1.6680 | 164.9418 | 12.9508 |
|  | Eq. 6 | 0.9904 | 0.2020 | 18.9176 | 4.3541 |
|  | Eq. 8* | 0.9944 | -0.0579 | 10.9877 | 0.9944 |

* represented the best basic model for estimating when using D & H as variable. Eq.4, Eq. 5, Eq. 6, Eq. 8 represented power, exponential, polynomial functions and the general model, respectively.

^a^ represented that through Duncan’s multiple range test, there was a significant difference (at 0.01 significant level) between Eq. 5 and other three models (Eq.4, Eq. 6, Eq. 8).

^b^ meant that under the condition of rounding four decimal places, the R^2^ of a power function (Eq.4) and the general model (Eq.8) were same, but the general model had lower RV and MSE, so we considered Eq.8 was optimal.
